# Personal and social determinants sustaining smoking practices in rural China: a qualitative study

**DOI:** 10.1186/1475-9276-13-12

**Published:** 2014-02-03

**Authors:** Aimei Mao, Tingzhong Yang, Joan L Bottorff, Gayl Sarbit

**Affiliations:** 1 Faculty of Health and Social Development, University of British Columbia’s Okanagan campus, Kelowna V1V 1 V7, Canada; 2 Centre for Tobacco Control Research, School of Medicine, Zhejiang University, Hangzhou 310058, China

**Keywords:** Smoking, Rural China, Social ecological model, Qualitative study

## Abstract

**Introduction:**

Tobacco use in China is disproportionally distributed among rural and urban populations with rural people smoking more. While there is a wealth of evidence on the association between tobacco use among rural people and their lower socio-economic status (SES), how social structural factors contribute to rural smoking is not well understood. Guided by a socio-ecological model, the objective of this study was to explore the personal and social determinants that play a key role in sustaining smoking practices among Chinese rural people.

**Methods:**

An ethnographic study was conducted in a rural area of Central Jiangsu, China. Participants (n = 29) were recruited from families where there was at least one smoking resident and there were young children. In-depth interviews and unstructured observations were used to collect data, which were then analyzed with an interpretive lens.

**Results:**

Although individuals had limited knowledge about the risks of smoking and lack of motivation to quit, social factors were in effect the main barriers to quitting smoking. Cigarette exchange and cigarette gifting permeated every aspect of rural family life, from economic activities to leisure pastimes, in family and wider social interactions. Traditional familism and collectivism interplayed with the pro-smoking environment and supported rural people’s smoking practices at the community level. Living in the rural area was also a barrier to quitting smoking because of the lack of information on smoking cessation and the influence of courtyard-based leisure activities that facilitated smoking.

**Conclusion:**

Development of comprehensive smoking cessation interventions in rural China needs to extend beyond an individual level to take into account the social determinants influencing smoking practices.

## Introduction

Although China has become the second biggest economy in the world, only after the United States, it is still a country in Stage Two of the tobacco epidemic, according to the widely cited Four Stage Epidemic Model established by Lopez [1]. This stage is characterized by a high prevalence of smoking in a context where the risks of tobacco smoking are not widely understood and tobacco control activities are generally not well-developed [1]. At the same time, China mirrors the global trend that smoking prevalence is disproportionally distributed toward socio-economically disadvantaged groups, with a higher rate of smoking among its rural population than its urban population [2,3]. Given that half of China’s 1.35 billion population lives in rural areas [4], the countryside represents a large pool of smokers.

Similar to the situation in other Asian countries, smoking is predominantly taken up by men in China, with more than half of adult men smoking, while less than 3% of women smoke [2,3]. National surveys in China have shown that men with a primary school background have the highest smoking rate [3] and that farmers and machine operators smoke at the highest rate among all occupations [2]. The differences in smoking prevalence between people from different backgrounds are often interrelated. The majority of people living in rural areas are farmers and farmers are usually the least educated among all the occupations [5,6]. While findings from previous research have added to the evidence linking disadvantaged socio-economic status (SES) with high smoking prevalence, how social structural factors contribute to smoking practice is not well understood.

It is sometimes hypothesized that people with higher SES are more interested in health matters and are accordingly more likely to give up smoking [7]. However, qualitative studies on social class and health have shown that health is considered equally important by all social groups [7]. Nonetheless, important differences have been identified among diverse SES groups. For example, people in lower SES groups usually endure more hardships in their lives and they may cope with the stress by smoking cigarettes [8-10]. Interestingly, a population-based research study in China showed a lower level of stress among rural people than their urban counterparts, despite the wide income gap between the two groups [11]. Coping with stress may not be the trigger for a higher smoking prevalence in rural China.

Although tobacco smoking is often framed as a personal choice and studies have explored personal factors prompting smoking, there are calls from researchers to explore broader social factors [10,12,13]. These researchers argue that solely emphasizing tobacco smoking as a personal lifestyle choice, while neglecting social-structural factors, supports a victim-blaming ideology. Quite often, individually-oriented interventions instruct people to be personally responsible for their health at a time when they are less capable as individuals to control their environment. Also, these interventions may not reach marginalized groups who are socially isolated, like people who live in rural and remote areas. There are suggestions that individually-oriented interventions should remain secondary to those addressing socio-structural factors [14,15].

Social networks play an important role in smoking practices in China because cigarette sharing and gifting is a 'social currency.’ Consequently, social networks act as both a facilitator for smoking initiation and a hindrance for smoking cessation (SC) [16-18]. However, researchers have reported contradictory findings related to the impacts of social networks on smoking status in rural China [16]. On the one hand, rural people are found to be more likely to quit smoking, supposedly due to their fewer social connections; on the other hand, there is higher smoking prevalence in rural China, for which social networks are found to be an important determinant [16]. Qualitative research has been suggested as a way to provide insight into the influence of complex social contexts on smoking in rural settings [10,16].

Despite higher smoking prevalence in rural China and the large rural population, studies on tobacco use in rural contexts are limited and have reported conflicting findings. There is an overall lack of public health resources in China and these limited resources are disproportionally allocated to favor urban areas [19,20]. Even experts and leaders engaged in public health suggest that the issues of tobacco use in rural areas may only be addressed after the issues in urban areas are resolved [16].

**Figure 1:**
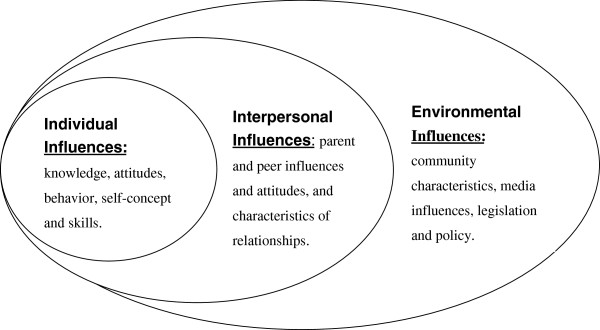
The socio-ecological model (based on Stokols’ theory).

Given the sparse data about tobacco use among rural people in China, there are challenges as well as opportunities for conducting research and guiding meaningful interventions. The objective of this qualitative study was to explore the personal and social determinants that sustain the high prevalence of smoking in rural China. A socio-ecological model (SEM) was used to guide this study. In his widely cited article on SEM, Stokols defined the term “ecology” as a study of “the relationships between individuals and their environments” [15]. Specifically, Stokols’ SEM divides health behavior determinants into three different levels, including: 1) individual factors, 2) interpersonal factors and 3) environmental factors, as illustrated in Figure 1. This study used an ethnographic approach because ethnography is founded on the belief that human behaviors are best understood in the fullest possible context [21]. This holistic approach therefore fits with socio-ecological perspectives. It is plausible that there are unique contextual factors that support cigarette use among rural dwellers and these factors need to be taken into account in designing SC programs.

## Methods

### Study setting

The study was conducted in central Jiangsu, China, traditionally one of the key areas of agricultural production in China. Located at the edge of Yangtze River Delta Economic Region, the most powerful engine of China’s economic development [22], Central Jiangsu has experienced rapid development since the economic reforms in the late 1970s. Income from off-farm activities, the participation of rural people in remunerative work away from their farmland, has replaced income from farming as the main income source for rural families. These more profitable activities include working in privately owned township and village enterprises or other private services, working as migrant workers in cities, running small family-based businesses and working as local craftsmen such as carpenters, bricklayers and painters [23]. In 2009, off-farm income, on average, constituted 78.5% of annual family monetary revenues in rural Jiangsu [24].

Following ethics approval from the University of Liverpool, the primary researcher (AM) went to her hometown, a village in Central Jiangsu, to conduct the fieldwork from October 2008 to August 2009. Participants were recruited from three villages and two countryside townships. Each village was inhabited by about 500–800 people while the two townships covered a population of around 80,000. In the past, the townships were exclusively occupied by people with urban status. However, with the recent inflow of farmers, the townships are now a mix of urban and rural dwellers. The village residents and those people who were officially registered as 'rural citizenship’ but lived in the townships were eligible for this study.

### Participant recruitment

Research findings suggest that families who are expecting children or who have young children are more likely to raise issues related to smoking (e.g., to protect infants and young children from second-hand smoke) and smokers in these families tend to change their smoking practices [25-30]. Therefore, two criteria were applied in recruiting participants: 1) participants came from a family with at least one preschool child aged six years or younger; and 2) there was at least one resident who was currently smoking. Smokers, as well as non-smokers who lived with smokers, were invited to participate in the study. A combination of purposive and snowball sampling strategies was used to recruit participants. Purposive sampling is the technique in which the researchers select individual participants who would be most likely to contribute appropriate data in terms of relevance and depth [31]. In this study, the researchers paid attention to recruiting participants from various demographic and social economic backgrounds such as age, education, family income, children’s age, number of smokers, and degrees of home smoking restrictions. The diversity of the participants provided rich data on personal and environmental determinants affecting men’s home smoking and the establishment of smoking restrictions. Purposive sampling was concurrently carried out with snowball sampling, in which the participants were asked by the researchers to assist with the recruitment of other participants [32,33]. Initial participants were recruited by AM through her social connections in the community. These participants were then asked to provide information about the study to others in the community who met the recruitment criteria and could potentially contribute to the study.

### Data collection

In this study, AM connected with participants in their natural setting for a prolonged period of time. AM became an active participant and an observer as she focused on the social-cultural experiences and practices of the participants. In-depth, open-ended interviews with all participants were the primary method of data collection. A topic guide for the interviews was developed based on the purpose of the study and critical readings of relevant literature [34]. The guide covered four areas: 1) smoking practices in the homes; 2) knowledge about risks of tobacco smoking and attitudes towards smoking; 3) opinions on reasons an individual continued to smoke; and 4) strategies used by family members to restrict home smoking. Questions asked in the interviews included: “Please describe smoking behaviors in your home;” “What do you think about the impacts of tobacco smoke on the health of smokers and non-smokers?” “Which factors do you think help to keep smokers smoking?” “Which factors help smokers quit or reduce smoking?” “How does your family deal with smoking behaviors at home?” Family members who smoked were asked: “How do you change your smoking when there are non-smoking family members around you?” and “How do you feel about the restrictions on smoking in your home?” If there were no restrictions at home the smokers were asked: “Do you think it is necessary to restrict smoking at home? Why (or why not)?” A brief questionnaire was used to gather basic demographic information and family members’ smoking situations, which included age, education, occupation, family income, number of children in the household, children’s age, number of smokers in the household, and whether there were restrictions on home smoking. An honorarium of 50 RMB (1RMB = 0.16 USD) was offered to participants in recognition of their contributions.

Initially, two rounds of interviews were conducted with the participants. The second interview took place any time from one week to one month after the first interview, and aimed to supplement and clarify any ambiguities resulting from the initial interviews. Data collection and analysis were concurrent and the interview guide was dynamic and iterative [35]. The additional questions for the second interviews were informed by the first interviews and the concepts and themes from the initial analysis were tested in the second interviews. Therefore, the questions were different with different participants. For example, one mother might be asked: “Last time you mentioned that your husband did not smoke near your child. Can you demonstrate how far he was away from the child? I have found people have different ideas about what it means to smoke 'near the child’.” Another mother was asked: “You once helped your husband quit smoking. But some mothers said that their husbands deserved to smoke because they earned money for the family. What do you think of this belief?” Despite the value of the second interviews, preliminary analyses revealed much repetition of information between the two rounds of interviews. Therefore, after having finished two rounds of interviews with all 16 mothers of young children, who were the first interviewees, it was decided that one interview would suffice for the remaining participants. However, this decision did not affect the dynamic nature of the interview guide, as identifying and refining important concepts is a key part of the iterative process of qualitative research. Participants from the same family were interviewed separately. All the interviews were conducted by AM at the locations chosen by the participants, mostly at their homes. The interviews lasted from 30 to 90 minutes.

Direct, first-hand observations of daily life provided a supplemental source of data for this study. AM lived in the field and used unstructured observations to collect information about smoking behaviors and people’s reactions to tobacco smoking in various contexts. Unstructured observations are not guided by an observation guide and therefore enable the observer to exercise flexibility on observing activities relevant to the study [36]. As such, observations for this study were not limited to the behaviors of the participants, but also included those of other people in the community. AM recorded field notes of the events and phenomena relevant to the purpose of the study. These field notes were later included as part of the data for analysis. The ten months of fieldwork provided sufficient data for a comprehensive examination of the multiple factors that sustain high smoking prevalence in rural China.

### Data analysis

Once all the data were collected, the interviews and observation notes were organized and transcribed. A systematic and rigorous analysis of the data, guided by Stokols’ Socio-ecological Model [15], was undertaken to identify salient themes, recurring ideas or language, and patterns of belief that linked the people and settings together. The comprehensive initial analysis was followed by additional readings of the data to determine relationships between the concepts.

### Findings

Twenty-nine family members from 22 families were recruited, including 16 mothers of young children, four fathers, five grandmothers and four grandfathers. The 21 female participants were non-smokers while the eight male participants were smokers. The demographics and smoking status of the participants and their families are shown in Table 1. Notably, those individual family members who farmed, without engaging in other economic activities, classified their occupation status as “no job” (没有工作). This reflected their perceptions of how minimal farming contributed to their families’ economy. Also of interest is that no single family relied solely on farming for their livelihood. Two families ran businesses (i.e., a family-based shop and restaurant) without farming while the other 20 were engaged in both on and off-farm activities.

**Table 1:** Demographics and smoking situation in the participant families

**Categories**	**Number**
**Family level data**	22
Family income (RMB/year)	
>100000	4
50000–100000	4
20000–50000	13
<20000	1
Number of smokers at home	
1	7
2	15
Household restrictions	
No smoking allowed inside the house	3
Smoking allowed in certain rooms	12
No restrictions	7
Families with children*	6
≤one year old	17
> one year old	
**Individual level data**	29
Family roles (In relation to young children)	
Mothers	16
Fathers	4
Grandmothers	5
Grandfathers	4
Age (years old)	
Mothers and fathers (21–34)	20
Grandmothers and grandfathers (55–67)	9
Occupation	
No job (farming only)	11
Off-farm activities	18
Education	
Elementary school or below	6
Middle school	14
Senior high school or vocational school	7
College	2
University	0

1RMB = 0.16 USD. *A family with one child under one year old and the other child over one year old.

In total, 43 individual interviews were completed, including two rounds with mothers and one round with other family members. In general, findings from these interviews and the researcher’s observations suggest that smoking among men in this area was widespread. Although participants mentioned that some men around them had never smoked, they stated that every man smoked on certain occasions. Observations in the community supported these perceptions. Cigarette smoking was observed to be an important part of men’s gatherings and people who had claimed to have never smoked or to have quit smoking received offers of cigarettes and sometimes even smoked with their peers. While smokers constructed smoking as a personal pleasure for men and minimized the risks of smoking to their health, smoking was described by both smokers and non-smokers as a “must” for a man in the current social environment.

The determinants sustaining smoking practices were framed according to multiple levels of behavioural influence factors, which produced a hierarchy of the determinants of initiation and maintenance of cigarette smoking (Table 2). The following section will detail the personal, interpersonal and environmental determinants identified in the study.

**Table 2:** Factors sustaining smoking in different levels

**Level of hierarchy**	**The themes supporting smoking**
Personal determinants	Superficial knowledge about harms of smoking
	Benefits of smoking
	Lack of knowledge about quitting methods
	No immediate desire to quit smoking
Interpersonal determinants	Co-smoking as happy family time
	Limited or no restrictions on smoking at home
	Cigarettes as a normal gift for male family smokers
	The cost of smoking
Environmental determinants	Cigarette as a facilitator in off-farm activities
	Smoking as a leisure activity
	Normalized violation of smoke-free bans

### Personal determinants

#### Superficial knowledge about harms of smoking

Although all participants agreed with the statement 'smoking is harmful to health’ (吸烟有害健康), their knowledge about risks of tobacco smoking was limited to immediate physical reactions to tobacco smoke, including coughing, sneezing and irritation to the throat and eyes. While non-smokers presented these physical symptoms as harms caused by tobacco smoke, smokers tended to regard them as only temporary *discomforts* rather than *harms.* One father who smoked stated: “I don’t believe that smoking is harmful. I think no smokers believe that. Yes, coughing. We cough when we smoke. Can you say other harms? Nothing else.” When asked to list the specific harms of tobacco smoke, two thirds of the participants acknowledged: “I only know that smoking is harmful to health. I know nothing else.”

#### Benefits of smoking

Smokers, and sometimes their non-smoking family members, listed a number of benefits of smoking, such as killing time, coping with stress, being part of a group, and enjoying the feeling of happiness or relaxation. Younger smokers tended to associate their smoking with their jobs. One father, who was a taxi driver, said: “I feel terribly sleepy at 3 and 4 in the early morning when I am at work. Smoking is the only way to keep me awake”. Some smokers said that smoking could make their brain “work fast” so that they could solve problems in their work. One grandfather who was a carpenter said that his smoking helped him to do the calculations required in his work.

#### Lack of knowledge about quitting methods

Local people believed that quitting smoking was entirely up to the smokers and that other people could do little to help with the quitting process. They had never heard of SC aids, such as nicotine replacement therapy (NRT) and counseling services. None of the smokers had ever sought external help in their previous attempts to quit smoking. However, they used such things as chewing gum, nuts, and sunflower seeds to help them fight their cravings when they were quitting smoking. Failures in quitting were often attributed to lack of determination, as one non-smoking mother said of her husband’s failure, “I think it was because he didn’t really want to quit. We all know the old saying 'Nothing is too difficult if you put your heart into it.’”

#### No immediate desire to quit smoking

At least half of the participants explained that men would usually temporarily quit smoking before or during their wives’ pregnancies and resume to previous levels by the time their children were old enough to go to kindergarten. Smokers and their family members talked about quitting smoking “someday”; however, plans for quitting were often in the distant future.

Older smokers explained how hard it would be for them to get rid of the old habit, as one grandfather said: “I have smoked for more than 40 years. If you ask me not to eat today, I can do it; if you ask me not to smoke today, I can’t do it.” The younger smokers described quitting smoking as “impossible” (不可能), “impractical” (不现实), explaining that “In today’s society, who doesn’t smoke?” Non-smoking family members supposed that only severe disease may trigger smokers to quit, “As long as they are healthy, they will continue to smoke.” One grandfather participant, during his interview with the researcher, mentioned his intention to quit smoking in the next month. However, the researcher saw him smoking on the street two months later. He laughed and acknowledged that he had no intention to quit smoking at present because he was healthy.

### Interpersonal determinants

#### Co-smoking as happy family time

In the 15 families where there was more than one smoker, it was common that the smokers smoked together. This co-smoking was regarded by both smokers and non-smokers as a way to build and maintain harmonious family relationships. So, non-smoking family members usually did not interfere with co-smoking behaviors. For example, a grandmother was unhappy with the co-smoking between her husband and his brother, but acknowledged that the co-smoking enhanced their brotherly relationship, “These two have never got red face[angry]to each other. Every morning his elder brother comes here, and the two sit there. Then you give me a cigarette; I give you a cigarette, and they smoke and chat. They get along very well.” In another family, the father and his adult son were smoking together when the researcher was conducting the interview with the mother participant in their home. She revealed that the two smoked every evening and that she did not want to intervene with her husband’s smoking because “I don’t want to destroy the atmosphere there. Also, his father will be unhappy if I say something against smoking because he is smoking, too.”

#### Limited or no restrictions on smoking at home

In the local rural area it was normal practice for people to leave their house doors open during the day if there were family members at home. The researcher observed the common phenomenon of home smoking, even in the families with young children. However, participants from 15 of the 22 families in this study reported having some restrictions on smoking in their homes. In these families, most of non-smoking women had successfully kept their bedrooms a smoke-free place by restricting their husband’s smoking there. These women expressed negative attitudes towards smoking, using negative terms when talking about the smoking situations in their home, such as 'disgusting” 'annoying”, 'irritating”, etc. However, only three families had a complete restriction on indoor smoking and the non-smoking women in other families expressed powerlessness in imposing a complete smoking ban. In the local areas, the senior generations were referred to by the juniors as shang-ren (上人), meaning the superordinate. It was difficult for junior women to regulate senior family men’s smoking because “You can’t confront your shang-ren”. Direct confrontation of the seniors was perceived by local people as a misconduct of filial piety (the moral practices the juniors should have for the seniors).

It was quite normal in rural Jiangsu that young and middle aged family men worked in cities and only came home occasionally. These men were believed to deserve a smoke when they came home, as one mother said: “No one says anything to their smoking. They only come home once or twice a year.” Family members certainly wanted the returned men to enjoy happy family life and confronting the men’s smoking would destroy the pleasant reunion time.

#### Cigarettes as a normal gift for male family smokers

In the local area, cigarettes, along with alcohol, were the most common gifts that family members gave to their male relatives and friends. Family gatherings, like birthday parties, wedding ceremonies, roof beam ceremonies (上梁, a ceremony for putting up the roof beam on a new house which is still under construction), and funeral gatherings, were consistently associated with smoking. For example, one neighbor of the researcher bought 30 cartons of cigarettes (10 packs/carton and 20 cigarettes/pack) for a birthday party which was attended by about 200 relatives and fellow villagers.

Cigarette gifting was particularly important for family juniors who were expected to show filial piety to senior generations or family elders. For example, one woman recounted that every time she and her husband visited her parents, she would remind her husband to bring cigarettes to her father who smoked. Another woman said that she bought her father cigarettes all year round as her way to thank him for helping with childcare.

Non-smoking family members did not have difficulty reconciling cigarette gifting with their negative attitudes towards smoking. According to them, their practice of cigarette gifting did not mean their support for family men’s smoking, “If I don’t give him cigarettes, he would buy them himself. My giving doesn’t affect his smoking.” The gifted cigarettes were almost always the 'haoyan’ (好烟), the more expensive cigarettes, and these cigarettes were believed to be less harmful to the health of the smokers than the cheaper ones the smokers usually consumed.

#### The cost of smoking

All the non-smokers believed that the money spent on cigarettes was a waste. However, for most of the families, the cost of cigarettes did not seem to have a significant impact on family life. One mother listed her husband’s expenses on smoking, “On average, he smokes up to 500 RMB a month….His salary is 2000 a month, and mine is 1000. Together we have 3000…. Right now we can manage our life because we don’t pay for our meals in our wider family [the extended family]” Another woman downplayed the financial burden of her father’s smoking, “My mum and I always persuade my dad to smoke less and save the money for food and clothes. It is not that he is short of food or clothes, but he can buy more and better clothes and food if he smokes less.” Apparently, the improved economic conditions in the past three decades meant that expenses of smoking did not affect the basic needs of the rural families.

### Environmental determinants

#### Cigarette as a facilitator in off-farm activities

Off-farm economic activities were the most important source of wealth for every family and cigarettes played an important role in those activities. This was prominently reflected in two ways: cigarettes as a type of payment, and cigarette gifting and sharing as a tool to establish and strengthen economic ties.

The use of cigarettes as a bonus payment to male employees was a common practice in the local private enterprises and services. Generally, male employees, even the non-smokers, got packs of cigarettes on a daily or monthly basis. For example, as the local bus companies had been privatized, all the bus drivers received two cartons of cigarettes from the owners of the buses at the beginning of each month. Among local craftsmen hired by individual families, it was a common practice for the craftsmen to receive a pack of cigarettes each day from their hosts. The offers of cigarettes were only for male employees and women employees, all of whom were perceived as non-smokers, did not receive additional payments in any form for comparable work.

The local people called cigarettes “the name card” (名片), or “the introduction card” (介绍信). Those who ran businesses remarked that “No cigarettes, no business.” Not only were men engaged in cigarette exchanges with business partners or customers but non-smoking women also took part in the practices. A non-smoking mother who helped with her family business of selling cement said that she offered cigarettes to the customers when her husband was not in the shop.

The jobs the rural people took on were usually short-term, and the end of these positions often coincided with the end of the Chinese Lunar Year, which usually falls in late January or early February in the Gregorian calendar. As a result, the Chinese New Year became a busy time for rural people to look for new jobs for the following year. Every man, whether a smoker or not, as well as non-smoking women, carried packs of cigarettes to visit local private employers or the labor contractors for projected jobs. Even those who were on a renewed contract were also involved in cigarette gifting in order to strengthen the relationship with their employers.

#### Smoking as a leisure activity

There were almost no public entertainment facilities in the countryside. Collective smoking in the local area happened in people’s houses rather than in public places and was part of men’s pastime activities. It was a common scene in the local area that on a sunny day several men drank tea and smoked cigarettes while chatting in the courtyard of a house.

Playing Mahjong (打麻将) is a favorite pastime in China. It is a table game with four players and is played for fun and money. For many of the local people, it was their only entertainment. Smoking was the usual companion for male players, “Every man smokes when they play Mahjong.” In addition, playing Mahjong was often a trigger for relapse and an obstacle for quitting smoking, as one grandfather who smoked said: “I find it is impossible for me to quit smoking. You can do that unless you don’t play Mahjong. But what else can you do except play Mahjong?”

#### Normalized violation of smoke-free bans

Smoking was commonly observed in some places supposed to be smoke-free. For example, kindergartens were designated to be smoke-free according to national regulations. However, the researcher found cigarette stubs both outside and inside the kindergartens in the two townships. The 'no-smoking’ signs were visible in almost every public vehicle but drivers often smoked inside the vehicles when they were waiting for the next turn at the end of a round. There were also cigarette stubs inside local small hospitals.

People accepted cigarette smoking in these designated 'no-smoking’ areas and regarded it as a countryside feature. For example, one mother participant who was a kindergarten teacher talked about the fact that no information on risks of tobacco smoke was delivered to children and parents in her school: “No one pays attention to the smoking problems in countryside kindergartens. Maybe urban kindergartens give the message to their children.” The head of her kindergarten showed surprise when she heard of the researcher’s study on tobacco control, saying: “Smoking? Isn’t it quite normal? Why control it?”

## Discussion

The findings from this study detailed the complex array of personal and social determinants that underpinned smoking practices in rural China. At the individual level, rural smokers advocated for the benefits of smoking while demonstrating an indifference to quitting. This lack of motivation to quit smoking may be associated with another important finding – the fact that rural smokers had minimal knowledge about the health risks of tobacco smoking. They also lacked skills and information on quitting methods. Viewed from a socio-ecological perspective, the absence in rural China of health education programs related to SC interventions has likely contributed to the low levels of knowledge and interest in SC.

Although the lack of motivation to quit smoking was a barrier at the personal level, the most important barrier to quitting smoking in contemporary rural China was the enhanced social currency of cigarette smoking. Two recent studies also showed the pervasiveness of cigarette exchange and cigarette gifting in rural China [17,18]. While these two studies focused on cigarette gifting and exchange between a family and its outside environment, our study was the first to reveal the implications of cigarette gifting and exchange to intra-family relationships and to the economic prosperity of the family. Smoking and cigarette exchange appeared to be highly integrated into family life in the local rural area. Despite negative attitudes towards smoking, non-smoking family members were caught in a dilemma between supporting and intervening in family men’s smoking [37]. Although they agreed that smoking was bad for health, their actions related to limiting men’s smoking was restricted by culturally framed role expectations to maintain family relationships and economic security. Maintaining a harmonious family relationship was viewed as paramount and individual family members were expected to put the family’s interests over their own interests in order to achieve family harmony and prosperity.

Unlike previous reports that farmers were isolated from the outside world [16], this study revealed active social interactions between rural people and their fellow villagers as well as the wider world in the expanding market economy. Researchers have described the significance of networks or Guangxi (关系) in private economic activities in a developing economy characterized by an imperfect legal and financial system [38]. This study found that, due to the dominance of informal and relationship-based contracts in the local area, employment was unstable, posing threats to both employers and employees. Strengthening networks through cigarette gifting was important to secure employment for employees and particularly for job seekers. For the local small business owners, networks could help them ease financial constraints and provide needed contracts to survive the increasingly competitive but partially marketized economic environment. Others have warned that as rural China continues to prosper, a significant portion of new wealth is likely to be spent on gifts of cigarettes, if no effective interventions are developed [17].

Although not directly examining the rural–urban inequality, this study described some of the underlying factors contributing to sustained smoking that are particular to rural China. Poorer infrastructure in rural areas limited people’s choices for diverse pastime activities, giving way to smoking-associated activities. A review has found an urban–rural division in the implementation of nationwide tobacco-related policies in developing countries including China [39]. While countries may be quite effective in enforcing no-smoking rules and regulations in towns and cities, due to vast urban–rural differences in education, health care, knowledge, and state monitoring, the no-smoking rules and regulations may not be as effective in small towns and villages, where tobacco-related laws are often overlooked [39]. One interesting finding in the study was that not only rural people ignored smoke-free regulations but they also identified smoking as a symbol of the rural class. A social environment in which smoking is very common, and in a sense self-evident, may easily facilitate its continuance without disruption [40]. The internalization of smoking as part of rural life may have further contributed to the local people’s lack of motivation to change the current smoking situation and their indifference to non-smoking regulations.

As an integral part of a socio-ecological perspective, understanding the role gender identities and gender relations play in determining health behavior is essential [41]. Gender norms and roles influence attitudes and behaviors in many areas, including relationships, parenting, schooling, work and health practices [13,42]. Gender roles can also create economic and cultural pressures that affect the health of females and males differently [13,25,26]. Research has shown that men’s smoking is related to their masculine ideologies of independence, physical resilience to harmful substances and capacity to endure risk-taking [25,26,43]. This study indicated that in the expanding economic market in rural China, men’s role as family provider was supported by smoking. The study also showed the impacts of the traditional patriarchal power system on smoking-related family interactions. Numerous studies have shown the negative impacts of non-smokers on their family members’ smoking. However, this study showed non-smoking women, particularly young women, in Chinese families played a limited role in preventing or regulating family men’s smoking. Addressing barriers to quitting smoking at the individual, interpersonal and environmental levels is necessary for enhancing the effectiveness of SC programs in rural China.

### Implications for policy and research

This study identified multi-level and inter-related factors in supporting the high prevalence of smoking in rural China. The findings offer clear direction for future SC interventions in some parts of China where the economic development is similar to Jiangsu Province, such as other parts of eastern China and southern China. Personal level interventions are needed to improve the knowledge of rural people about the risks of tobacco use and thus increase their motivation to quit smoking. Educational programs are scarce in rural China and health professionals should take advantage of 'teachable moments’, such as the time around women’s pregnancy or the time of illness, to provide advice and support for SC. Studies with pregnant women and mothers of young children who were ill showed that non-smoking women played a role in reducing partners’ smoking shortly after interventions from medical professionals, although no long-term positive results were observed [27-30]. Further research should be undertaken to develop similar interventions that can be integrated into general clinical practices, in obstetrical departments and pediatric departments, and these interventions can be applied repeatedly to sustain effectiveness. Men should be involved to guide the development of these interventions which are framed by gender relationships in Chinese families [37,44].

According to socio- ecological perspectives, individual-level or family-based strategies may only be effective if the pro-smoking social environment is simultaneously addressed. As shown in the study, traditional values of familism and collectivism contribute to smoking. However, these values become smoking facilitators only in specific social environments. A large scale study in California showed that 52.5% of Chinese smokers quit smoking after they went to the USA, although only 17% of smokers in China quit smoking [45]. The significant difference in the quitting rates provides support for denormalization of smoking practices as an effective way to curb tobacco use.

Addressing the social practice of cigarette sharing and gifting is the key to reducing the high smoking prevalence in rural China. A mass media campaign called “Giving Cigarettes is Giving Harm” (送烟就是送危害) has been conducted in some cities [46]. However, no similar campaigns have been conducted in rural China. City-wide or community-wide campaigns against sharing and gifting of cigarettes can support local residents who do not want to be seen as inhospitable in a culture where the exchange of tobacco has positive cultural associations. Experiences from Project Quit Tobacco International showed that support from community groups can enhance an individual’s or a family’s efficacy to reduce smoking [47]. Given the more pervasive smoking at Chinese festivals, also reported in the study, community-based interventions can take advantage of Chinese festivals to reach more rural people.

Other strategies that hold potential at the population level include health education to correct the misconception that more expensive cigarettes are less harmful to health than cheap ones and the use of mandated text and graphic warnings on all cigarette packages to make cigarettes culturally inappropriate for gifts [17,18]. The World Health Organization strongly recommends taxation on tobacco products and smoking bans in public places [48]. However, implementation of taxation and smoking bans in rural China would be challenging because of the perceived economic gains associated with smoking and the blurring line between public places and private premises. Further research is needed to explore effective ways to enact these policies in the rural context.

Another important issue for controlling smoking in rural China is to balance the distribution of health resources between rural and urban populations. Improved infrastructure in rural areas may help people engage in healthier activities and move away from courtyard-based collective smoking practices. Directing health resources to tobacco control programs in rural areas would create a supportive social environment for SC.

### Limitations

There are limitations with this study. First, given the small number of participants, findings from this study cannot be generalized beyond the local area of the study. Second, socio-economic variability in the vast rural areas of China also limits the findings’ generalizability. Specifically, this study took place in one of the most affluent provinces in China and the socio-economic conditions of the participants in this study may not be similar to those of rural people living in poorer areas in western China or inland China. Impacts of smoking on family budgets and family members’ attitudes toward smoking among the participant families may be different from those of families in less developed areas. Third, some individuals may not have been fully forthcoming in their responses, although the long-term fieldwork and triangulation of data resources have significantly enhanced the credibility and trustworthiness of the findings.

## Conclusion

This study revealed unique features about tobacco use in rural China in a post-reform era. There are a multitude of interrelated factors contributing to the high smoking prevalence in rural China, indicating the importance of multi-dimensional SC programs. This study calls for urgent action on smoking in rural China, rather than ignoring the issue until smoking in urban areas has been resolved. Economic development in rural China is not resulting in a decline in tobacco use, as predicted by Lopez’s theory of tobacco epidemic [1], but instead leading to more prevalent smoking. Tackling smoking in rural China will be challenging due to the complicated personal, social and environmental barriers. This study has provided an initial look at these barriers, but more studies are needed to inform evidence-based SC interventions.

## Competing interests

The authors declared no potential conflicts of interest with respect to the research, authorship, and/or publication of this article.

## Authors’ contributions

AM undertook data collection and analysis and drafted the manuscript. TY, JB and GS helped to refine presentation of the analysis and contributed to development of the manuscript. All authors read and approved the final manuscript.
